# Rhetorical Candour: When and How to Say It Straight in Your Academic Manuscript

**DOI:** 10.5334/pme.2269

**Published:** 2026-03-13

**Authors:** Lorelei Lingard

**Affiliations:** 1 Centre for Education Research & Innovation, Schulich School of Medicine & Dentistry, Western University, Medical Sciences Building, Suite 100 London, Ontario, N6G 2V4, CA

## Abstract

This *Writer’s Craft* explores *rhetorical candour*: the art of saying what you mean and saying it straight, while remaining scholarly and credible. Through control of modality and sentence structure, writers can avoid the habitual academic hedge so that their work is nuanced where necessary and plainspoken where effective.

In the writer’s craft section we offer simple tips to improve your writing in one of three areas: Energy, Clarity and Persuasiveness. Each entry focuses on a key writing feature or strategy, illustrates how it commonly goes wrong, teaches the grammatical underpinnings necessary to understand it and offers suggestions to wield it effectively.

*The great enemy of clear language is insincerity. When there is a gap between one’s real and one’s declared aims, one turns as it were instinctively to long words and exhausted idioms, like a cuttlefish spurting out ink*.

-George Orwell [1]

You probably know the feeling. You write some sentences that seem clear, but then, out of respect for nuance or a sense of caution, you add a wee hedge: *may, might, perhaps*, or *to some extent*. Then you fold in another hedge, and another. Before long, your paragraph feels like it’s been written from nowhere by no-one: it’s so polite and non-committal that your reader can’t tell what you actually think.

Hedging is not, in itself, a flaw. On the contrary, it performs two key functions in scholarly writing. First, it signals your degree of certainty about, and therefore your commitment to, a claim [2]. Second, it softens assertions that might otherwise threaten writer or reader ‘face’ [3]. We need hedging to express a stance and relate to our readers. However, when hedging becomes habitual, it shifts from rhetorical to reflexive, potentially alienating readers who are left unsure where you stand, or whether you stand anywhere at all. In this *Writer’s Craft*, we’ll explore rhetorical candour: the art of knowing when and how to say it straight.

## Rhetorical Candour

“Rhetorical candour” is an antidote to the habitual hedge. It represents an advanced tool for writers to calibrate their stance so that their commitment is commensurate with the evidence. The term contains echoes of two neighbouring ideas. *Intellectual candour* [4] describes an educational approach based on the courage to reveal vulnerability while retaining credibility in teaching and learning. *Radical candor* [5] describes a business leadership approach that combines direct challenge with personal care to promote teamwork. Rhetorical candour similarly asks us to inhabit tensions in academic writing: to be committed without bravado, tentative without evasiveness. It is an intentional stance that treats clarity – at the right time and in the right measure – as both an intellectual and an ethical good.

**Figure 1:**
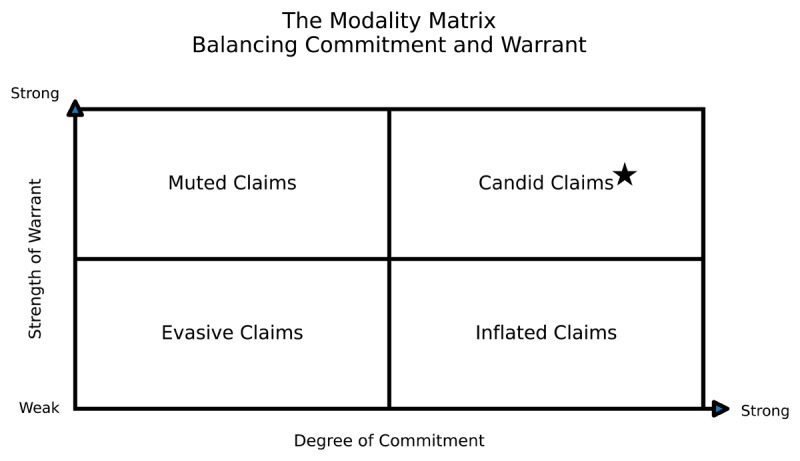
“The Modality Matrix” classifies scholarly claims according to the axes of Warrant (strength of evidence and reasoning) and Commitment (degree of authorial certainty and directness).

Rhetorical candour emerges from the interplay between two axes: *commitment* and *warrant*. Commitment, from Hyland’s model of stance in scholarly writing [2], refers to the degree to which the writer portrays certainty in the claim being presented: high commitment is signaled by minimal hedging while low commitment is characterized by increased hedging. Warrant, from Toulmin’s model of argumentation [6], refers to how well the evidence and reasoning justify the claim. Their intersection forms what we can think of as a Modality Matrix (Figure 1), which writers can use to help calibrate their hedging appropriately.

Used as a heuristic, the modality matrix can help writers calibrate their hedging and identify moments appropriate for rhetorical candour. Muted Claims have strong warrants but minimized authorial commitment: e.g., the claim, “Medical school may involve stress and high workload”, is based on a sufficiently strong warrant that the hedge “may” seems unnecessarily tentative. Evasive Claims lack both warrant strength and commitment: e.g., if we write “In some situations, gender may potentially have implications for training experience”, we’ve layered in multiple hedges (“in some situations”, “may”) and managed not to commit to anything. Inflated Claims are overcommitted relative to the low strength of the warrant: e.g., the statement “Clinical performance assessment is fundamentally flawed” is boosted by the adverb “fundamentally”, which could come across as an overstatement. Candid Claims match strong warrant with high authorial commitment: in such situations, minimally hedged or even unhedged writing is appropriate. For example, we know enough about health resource constraints in rural environments that we can write “Canada’s rural healthcare workforce is stretched” without adding hedges like “it may be the case that” or “perhaps”.

The goal is not to purge hedges but to *control* them. We want to use them when they reflect an appropriate calibration of warrant and commitment. To illustrate, Box 1 offers four versions of the same sentence under conditions of a strong warrant.

**Box 1 Modality choices under conditions of strong warrant**

*Warrant context: Multiple empirical studies, learner narratives, and educational theory converge to suggest that writing plays an important role in the development of clinical reasoning among trainees. Given this warrant, the following sentences illustrate different modal expressions of the same underlying claim*.

**Muted claim** (under-committed relative to warrant)

It is possible that when AI scribes handle written documentation, clinical clerks may lose an opportunity to practice reasoning through writing.

**Evasive claim** (non-committal despite warrant)

There may be complex implications of AI scribes for how clinical clerks engage with written documentation and clinical reasoning.

**Inflated claim** (over-committed relative to warrant)

When AI scribes handle written documentation, clinical clerks inevitably lose their ability to develop clinical reasoning.

**Candid claim** (proportionate to warrant)

When AI scribes handle written documentation, clinical clerks lose an opportunity to practice reasoning through writing.

Box 1 shows how the same statement can sound very different depending on how much responsibility the writer takes for it. In the Muted version, the claim is still there (“clinical clerks may lose an opportunity”), but it is softened by layers of hedging (“it is possible”, “may lose”) that dial back commitment even though the warrant is strong. The Evasive version recedes further, signaling relevance without actually stating a position (“how clinical clerks engage”). The Inflated version goes in the opposite direction, boosting the claim (“inevitably”) beyond what the warrant can support. Only the Candid version is well-calibrated: it says something clear, bounded, and arguable, and it does so in a way that fits the strength of the warrant.

Now that we understand how and why we calibrate our hedges in academic writing, let’s return to the problem we started with: habitual hedging. Habitual hedging happens when writers end up in the Muted or Evasive quadrants of the matrix when the warrant strength doesn’t require it. Why tiptoe around a claim when the warrant is strong? One reason is politeness [7]. Hedging does important social work: it signals respect for other researchers, leaves space for disagreement, and helps writers navigate contested terrain [8]. Writers who continue to hedge even when the warrant is strong may be doing so because taking a clear position can feel risky, confrontational, or impolite. Another reason is convention: the expectation of neutral, disinterested scientific writing promotes conservatism, which can make strong commitments feel immodest and subjective. Culture also influences hedging: comparative analyses of academic writing suggest that English writers “tone down” their claims, communicating lower commitment relative to writers from other countries [9]. Consequently, for scholars writing in English as an additional language (EAL), hedging can be particularly difficult to calibrate. While resources exist to assist EAL writers, they tend to emphasize diluting the certainty of claims without reference to the strength of warrants [10]. But when writers are generally encouraged to “be modest”, “avoid bold, arrogant claims”, and “protect your ideas from undue criticism” [11], this can produce habitual hedging -- writing that lacks stance, writing from nowhere.

## Achieving rhetorical candour

This Writer’s Craft isn’t just a lesson in modality. It is a call for rhetorical candour in our scientific writing. That means writing clearly and boldly *when the warrant is strong*. Writing this way requires leaving the comfort zone of the Muted and Evasive quadrants for the unequivocal zone of the Candid quadrant.

In order to write with rhetorical candour, we need to recall how modality works at the grammatical level [12]. Verbs are a primary means for tuning between tentativeness and certainty. Auxiliary verbs such as *could, would, should, may, might, must* combine with main verbs: e.g., “Medical schools *could* formalize anti-violence training” is more tentative than “Medical schools *should* formalize anti-violence training.” Verbs can contain modality without adding an auxiliary: e.g., “suggest” is more tentative than “conclude” or “advocate”. Adverbs are another modality mainstay: e.g., *significantly, clearly, usually* boost certainty, while “*possibly, apparently, arguably*” limit certainty. Writers can layer in multiple hedges, or leave sentences unhedged. Consider the following sentences, ordered from most tentative to most certain, where the hedges and **boosts** are visually marked:

Layered Hedged: There is concern that current political shifts may threaten some equity initiatives.

Hedged: Current political shifts may threaten equity initiatives.

**Boosted**: Current political shifts **undoubtedly** threaten equity initiatives.

Unhedged: Current political shifts threaten equity initiatives.

Notice that the strongest commitment is not the boosted version, but the unhedged one, which presents the claim as a statement of fact.

Sentence structure is also a key tool when you’re trying to position your work purposefully within the modality matrix. Longer, complex sentences are structurally amenable to hedging, while shorter, simple sentences offer the opportunity for boldness. Complex sentences build in hedging when the subordinate clause tempers the main clause. Consider the following example:

While some of the findings described here may be specific to the UK, student bullying is an issue of concern for higher education in many countries.

Here, the subordinate clause hedges the claim, situating it contextually. By contrast, a short, declarative sentence feels bold because it leaves no room for equivocating about the author’s position. Notice how the short, simple sentence in the following paragraph [13] feels plainspoken, a breath of fresh air:

“It would have been reassuring had we found many significant, inverse relationships between respondents’ training in research integrity and behaviors that may compromise the integrity of science. We did not.”

I suspect the writers of “We did not” may have been tempted to hedge a bit, perhaps by using an adverb like “Unfortunately” or by writing a more impersonal sentence like “Instead, our findings were more complex”. Rhetorical candour is achieved in this example both by the lack of hedging and by the first-person point of view combined with active voice: it’s not only an unhedged claim, it’s an unhedged claim spoken by “We” the researchers.

## Enhancing rhetorical candour

When we go looking for places where we might be more candid in our writing, they’re everywhere. Even in writing that is already very good! In the following published paragraph [4], I’ve toned down the hedges to strengthen rhetorical candour:

Reflection demands that learners take risks in making judgements about their own performance. There is further risk in communicating these judgements to supervisors, who often adopt a have the combined role of assessor and supporter/developer. Previous studies indicate that Learners find these processes of self-exposure daunting, fraught and, at times, the opposite of self-serving in a competitive and high-stakes industry [8,9]. These risks may be are real: fallibility and vulnerability are not viewed as desirable characteristics within the health professions education culture [10].

Here’s an example from my own published writing [14], where I’ve toned down the hedges to be more candid:

Much published research is dull, and dry; it may also be and impenetrable to readers. These problems likely contribute to a well-documented knowledge translation challenge in health research.

The first sentence in my example is already hedged: “Much published research”, not “All”. So why did I add “may also be”? Similarly, the verb “contribute” is already a lexical hedge: it signals ‘part of’ the knowledge translation challenge, not the whole challenge. Looking back, the extra, layered hedges feel more like habits than necessary nuance. As these examples demonstrate, candid writing doesn’t mean *no* hedges; it means proportionate hedges.

If you suspect habitual hedging may be a feature of your writing, try using the Modality Matrix as a diagnostic tool. Reread a section of one of your own manuscripts. Take note of the verbs and adverbs that temper your claims. Notice how you’re using sentence structure to hedge. Ask yourself: is each hedge serving accuracy (commitment calibrated to warrant), or anxiety (convention and politeness)? Map your claims into the matrix: which quadrants are you occupying? Then, experiment with revision (Box 2). Remove layers from a Muted or Evasive claim. Experiment with a Candid claim by letting a sentence stand unhedged. Feel the discomfort of exposure—that is where candour begins.

**Box 2 Spotting opportunities for rhetorical candour**

Use these prompts to revise your writing:

- Where is the warrant strong enough to support a clearer claim?
- What would this sentence sound like if I removed one hedge? Two?
- What is the smallest increase in commitment that would make this claim clearer, not louder?
- If I’m hedging for politeness, what am I trying to protect—my reader, myself, or the argument?

These are often moments where candid claims are possible.

## Summary

The impulse to hedge is understandable, but hedging where you don’t need to undermines clarity and credibility. Rhetorical candour is an invitation to own your stance—to replace habitual hedging with calibrated hedging and to make clear, candid claims where the evidence warrants it. It doesn’t mean abandoning caution or politeness; it means noticing when those instincts are steering the writing more than the evidence is—and choosing, deliberately, to let the warrant carry the weight. Add rhetorical candour to your writing repertoire, but take care: ask trusted colleagues to help you refine your ability to judge when and how it strengthens your arguments. It takes clarity and courage to say what you mean, but it is, ultimately, a gesture of intellectual integrity.

## AI-use disclosure statement

ChatGPT 5o created a first draft of this manuscript on Oct 29, 2025. Initial prompting oriented it to the tone and genre of the Writer’s Craft series, after which I uploaded a Powerpoint file from a workshop I had given on Rhetorical Candour and requested that it be shaped into a Writer’s Craft paper. I then interacted with ChatGPT around sections of its draft; e.g., correcting its summary of the main idea, redirecting it to replicate precise language in the Powerpoint document and requesting edits of particular sections. Once a workable draft was achieved through these interactions, I exported it into Word and edited substantially to remove repetition, add refinements (such as additional examples) and reorganize sections. Following constructive peer review commentary, I interacted again with ChatGPT on Jan 27, 2026 to brainstorm more conceptually precise labels for the two axes in Figure 1. I selected the best of the options suggested, refined this language, verified the theoretical literature underpinning it, revised the language throughout the paper to reflect this refinement, and recreated Figure 1 with ChatGPT’s assistance. I take full responsibility for all material in the paper. The full chatlogs are available upon request.

## References

[1] George O. Politics and the English Language. Horizon; 1946.

[2] Hyland K. Writing without conviction? Hedging in science research articles. Appl Linguist. 1996;17:433–54. DOI: 10.1093/applin/17.4.433

[3] Myers G. The pragmatics of politeness in scientific articles. Appl Linguist. 1989;10:1–35. DOI: 10.1093/applin/10.1.1

[4] Molloy E, Bearman M. Embracing the tension between vulnerability and credibility: ‘intellectual candour’ in health professions education. Med Educ. 2019;53:32–41. DOI: 10.1111/medu.13649 30192024

[5] Scott K. Radical candor How to get what you want by saying what you mean. Pan Books; 2019.

[6] Toulmin S. The Uses of Argument. Cambridge University Press; 1958.

[7] Brown P, Levinson SC. Politeness: Some Universals in Language Usage. Cambridge University Press; 1987. DOI: 10.1017/CBO9780511813085

[8] Lingard L, Watling C. The Academic Hedge, Part II: Getting Politeness Right in Your Research Writing. In: Story, Not Study: 30 Brief Lessons to Inspire Health Researchers as Writers. Innovation and Change in Professional Education, vol 19. Springer, Cham; 2021. DOI: 10.1007/978-3-030-71363-8_22

[9] Salager-Meyer F. Scientific discourse and contrastive linguistics: Hedging. European Science Editing. 2011;37:35–37.

[10] Kojima T, Popiel HA. Effective Use of Hedging in Scientific Manuscripts: Advice to Non-Native English-Speaking Researchers. J Korean Med Sci. 2023 May 1;38(17):e152. PMID: 37128879; PMCID: PMC10151619. DOI: 10.3346/jkms.2023.38.e152 37128879 PMC10151619

[11] Refnwrite. Hedging Techniques in Academic Writing with Examples. Accessed Feb 4, 2026; 2024. https://www.ref-n-write.com/blog/hedging-techniques-in-academic-writing-with-examples/.

[12] Lingard L. The academic hedge Part I: Modal tuning in your research writing. Perspect Med Educ. 2020;9:107–110. DOI: 10.1007/S40037-019-00559-Y 31953653 PMC7138759

[13] Anderson M, et al. What Do Mentoring and Training in the Responsible Conduct of Research Have To Do with Scientists’ Misbehavior? Findings from a National Survey of NIH-Funded Scientists. Academic Medicine. 2007;82(9):853–860. DOI: 10.1097/ACM.0b013e31812f764c 17726390

[14] Lingard L, Watling C. The writer’s voice repertoire: Exploring how health researchers accomplish a distinctive ‘voice’ in their writing. Med Educ. 2024;58(5):523–534. DOI: 10.1111/medu.15298 38233970

